# Spontaneous fetal reduction in triplets and prolongation of twin pregnancy for 111 days as an outpatient: a case report

**DOI:** 10.1186/s13256-021-02952-w

**Published:** 2021-06-21

**Authors:** Davis Rubagumya, Munawar Kaguta, Ernie Mdachi, Muzdalfat Abeid, Hussein Kidanto

**Affiliations:** 1 Department of Obstetrics and Gynecology, The Aga Khan Hospital-Dar es Salaam, PO Box 2289, Dar es Salaam, Tanzania; 2 Department of Family Medicine, The Aga Khan University-Dar es Salaam Campus, PO Box 38129, Dar es Salaam, Tanzania; 3 Family Medicine-Premier Care clinic-Masaki, PO Box 220, Dar es Salaam, Tanzania

**Keywords:** Triplets, Spontaneous fetal reduction, Delayed interval delivery, Multiple gestation, Case report

## Abstract

**Background:**

Multiple gestation has been on the rise because of advancement in assisted reproductive technology. Triplet pregnancy is associated with fetal loss and preterm birth as its major complications. Spontaneous triplet pregnancy is rare. In the case of fetal loss, delayed interval delivery has been used to achieve delivery of the retained fetuses. There is no common approach to delayed interval delivery.

**Case:**

A 31-year-old East African lady with spontaneous triplet pregnancy presented to our institution at gestation age of 19 weeks with features of threatened miscarriage. One fetus was miscarried, and delayed interval delivery was done as an outpatient. At gestation age of 35 weeks, she delivered healthy twins by cesarean section.

**Conclusion:**

Delayed interval delivery improves neonatal outcomes of high-order pregnancy after fetal loss even in a resource-limited setting.

## Background

Multiple gestation means conceiving more than one fetus. Of late, multiple gestation has been on the rise because of the advancement in assisted reproductive technology. Incidence of spontaneous triplet pregnancy is estimated to be 1/7000 [1]. Presence of multifetal pregnancy requires extensive assessment and counseling during antenatal care [2]. The basic assessment in multifetal pregnancy involves determination of the gestational age and chorioamnionicity, and assessing for any congenital anomalies among fetuses [2].

Maternal–fetal risks and complications increase with multiple gestation [3]. Triplet pregnancy has more risks and complications compared with twin and singleton pregnancy [4]. Triplet pregnancy is complicated by fetal loss before gestational age of 24 weeks and preterm delivery before gestational age of 32 weeks [4]. Other complications include hypertensive disorders of pregnancy, *abruptio placentae*, placenta previa, fetal growth discordance, preterm premature rupture of membranes, twin-to-twin transfusion syndrome, twin anemia polycythemia sequence, cord entanglement, antepartum death, acute fatty liver, and postpartum hemorrhage [2, 4-6].

With increased risks and complications, several measures are taken in caring for multifetal pregnancy, such as pregnancy assessment with the use of ultrasound, provision of micronutrients and preeclampsia prophylaxis, screening for maternal anemia and gestational diabetes, monitoring for preterm labor and use of tocolytics when needed, use of antenatal corticosteroids for lung maturation in case delivery is anticipated before 34 weeks of gestation and magnesium sulfate when delivery is inevitable at gestation age between 23 and 31 weeks, selective fetal reduction, and delayed interval delivery [2, 5, 7].

Sharing of chorion or amnion increases the probability of complications in multifetal pregnancy [2]. Of note is the fact that gestational age at delivery is inversely proportional to number of intrauterine fetuses, and directly related to parity [8, 9]. Fetal loss can happen to all or one of the fetuses [10]. Fetal reduction is reported to improve outcome of the retained fetuses, especially in elective fetal reduction as compared with spontaneous [8, 11]. Improved neonatal outcomes after extending delivery time has been reported [12, 13]. This strategy is termed delayed interval delivery (DID) and was pioneered by Carson in 1880 [4]. DID seeks to avoid pregnancy termination and maintain the retained fetus for as long as possible [4]. For multifetal pregnancy in preterm labor, the expulsion of one fetus is reported to bring cessation of contractions [4].

There is no ideal algorithm for performing DID. Depending on the available resources, the following are considered:

(1) Candidate selection and acquiring informed consent; for high-order pregnancy at gestation age of < 24 weeks with spontaneous fetal loss due to preterm labor or cervical insufficiency. Complicated pregnancies such as ones with preeclampsia are contraindicated [12, 14].

(2) Exclusion of intraamniotic infection of the retained fetus. Evidence of clinical chorioamnionitis in the delivered fetus sac warrants provision of broad-spectrum antibiotics and DID [12].

(3) Pragmatic umbilical cord and placenta management. In spontaneous fetal reduction, avoid cord traction and leave the placenta *in situ*. Cord should be clamped, cut, and ligated as proximal to placenta insertion as possible [4, 12].

Moreover, for Rhesus-negative mothers, Anti-D immune globulin should be given post-delivery of the fetus [12]. Also, despite controversies, prophylactic antibiotics should be given especially when cerclage is considered [4, 12].

Last but not least is the insertion of cervical cerclage. Despite controversies, such as postprocedure chorioamnionitis, this is done when there is high index of suspicion for cervical incompetence [4, 12].

Discharge instruction should include explanation of danger signs, and antenatal follow-up should be regular.

Case reports and studies to evaluate effectiveness of different approaches to delayed interval delivery are needed for different regions with different resources.

## Case

We report a case of successful spontaneous reduction of triplets to twins. Mrs. L.K, a 31-year-old East African lady, gravida 2 para 1 living 1, with spontaneous triplets at 19^+5^ weeks of gestation from last menstrual period (LNMP), was admitted to our hospital complaining of lower abdominal pain associated with vaginal blood spotting. There was no history of trauma, fever,or headache, and her antenatal records were unremarkable. There was no family history of multiple gestations. Two years prior to the index pregnancy, she vaginally delivered a healthy baby at 41 weeks of gestation. Her physical examination was unremarkable, and speculum examination revealed blood within the vaginal vault with no active bleeding from the opened cervix. Obstetric ultrasound revealed triplet dichorionic triamniotic live pregnancy corresponding to gestational age of 18^+2^, 18^+6^, and 17^+6^ weeks, respectively, without fetal anomaly, and her cervix was short and open at the internal os. She was prescribed a complete bed rest, intravenous ampicillin 500 mg every 6 hours for 5 days, and oral nifedipine retard 20 mg every 12 hours. Her vital signs remained stable, and on the second day postadmission, there was improvement with no vaginal leakage and significant reduction of lower abdominal pain; however, during the evening ward round, she started experiencing increase in the intensity of lower abdominal pain associated with vaginal spotting; therefore, 24-hour infusion of intravenous MgSO_4_ 4 g was prescribed. On third day postadmission, she miscarried a female fetus weighing 121 g with its complete placenta and membranes. Her lower abdominal pain subsided significantly, and there was no vaginal leakage on fourth day postadmission. Obstetric ultrasound on day 4 postadmission revealed dichorionic diamniotic viable twin pregnancy corresponding to gestational age of 19^+2^ and 18^+3^ weeks, respectively, and the cervix was 4.1 cm long with funneling at the internal os measuring 1.58 cm. Macdonald’s cervical cerclage was applied. Injectable progesterone 250 mg IM STAT dose and oral dydrogesterone 10 mg every 12 hours were prescribed. On fifth day postadmission, she had no complaints and oral nifedipine was stopped. She was discharged on sixth day postadmission after undergoing an ultrasound that revealed dichorionic diamniotic viable twin pregnancy corresponding to 19 and 18^+3^ weeks, respectively, and cervix 3.79 cm long with a closed internal os. Discharge medications included daily oral 162 mg capsule that contains ferrous fumarate, folic acid, and vitamin B12, and oral dydrogesterone 10 mg every 12 hours for 12 days. She was educated on danger signs prompting immediate evaluation as well as being scheduled for regular antenatal clinic visits.

At gestational age of 30^+2^ weeks, she underwent obstetric ultrasound as an outpatient client, which revealed dichorionic diamniotic viable twin pregnancy corresponding to 26 ^+3^ and 27^+5^ weeks weighing 907 and 1165 g, respectively, without fetal anomaly, and the cervix was 3.7 cm long with closed internal os. She had no complaints during this visit.

At gestation age of 34^+1^ weeks, she was seen at our antenatal clinic as part of her scheduled follow-up, and follow-up ultrasound revealed dichorionic diamniotic viable twin pregnancy corresponding to 30^+1^ and 32^+2^ weeks weighing 1586 and 2021 g, respectively, and the cervix was closed.

At 35^+1^ weeks, she presented at our hospital with preterm premature rupture of membranes. She had no fever, headache, or lower abdominal pain. Her physical examination was unremarkable apart from gushing fluid per vaginal on speculum examination. Fetal wellbeing was assessed with the use of cardiotocography, and the results were unremarkable. Emergency cesarean section was done whereby female twins in breech and cephalic presentation were delivered weighing 1.32 and 2.54 kg, with good Apgar scores, and they were transferred to Neonatal High Dependency Unit for observation. Cervical cerclage was removed intraoperatively. She fared well, and was discharged 72 hours post-delivery.

## Discussion

This case involved multifetal gestation that happened spontaneously. The reported incidence of spontaneous triplets is low [1], indicating that our case is rare.

Chorionicity does have a significant role in the outcome of multifetal pregnancy with reports suggesting that monochorionic and dichorionic triplet pregnancies have higher rates of poor perinatal and neonatal outcomes as compared with trichorionic pregnancy [15, 16]. Our client’s pregnancy was dichorionic triamniotic, which is always associated with risks involving; twin-to-twin transfusion syndrome, discordant growth, twin anemia polycythemia sequence, and increased chances of fetal loss. For our case, the fetuses had variable weights, and spontaneous fetal reduction happened, indicating the increase in risks with shared chorion, prompting extensive care in these cases.

Higher-order pregnancies are associated with complications, with fetal loss and preterm birth being major ones for triplet pregnancy [4]. In our case, spontaneous labor occurred at gestational age of 19 weeks, that is, before 24 weeks, and after thorough assessment of our patient, different measures were taken to stop labor without success. Interestingly, after miscarriage of one fetus, all of our client’s symptoms and signs subsided significantly, and this is similar with the observation that fetal reduction leads to cessation of labor [4]. Some case reports had shown spontaneous fetal reduction happening without any signs and symptoms of labor, and others even needed removal of cervical cerclage [4]. Other studies have shown retention of placenta, *abruptio placentae*, and postpartum hemorrhage as maternal complications in delayed interval delivery involving twins and triplets [14]. However, none of these happened in our case.

Our client was managed with antibiotics and synthetic progestogens in addition to Macdonald cervical cerclage, and extended time was 111 days. Her course of management was as an outpatient from day 2 post cervical cerclage to the time of cesarean section. Different studies have indicated controversies with cervical cerclage, even reporting it as the source of chorioamnionitis for the remaining fetus, hence prompting short duration of delayed interval delivery [12, 17]. Some case reports advocated inpatient management during the extended interval until delivery; however, some other case reports showed success with outpatient management. For cases that reported outpatient management, the hospital stay before discharge after loss of first fetus was no less than 1 week [4, 17].

## Conclusion

Delayed interval delivery is recommended in carefully selected cases and can be done even in limited-resource setting, and it is recommended for better neonatal outcome especially in these areas since caring for extreme preterm babies carries higher neonatal mortality rate.

## Data Availability

Available from the corresponding author upon request.

## Abbreviations

DID: Delayed interval delivery
g: Gram
IM: Intramuscular
kg: Kilogram
LNMP: Last normal menstrual period
MgSO_4_: Magnesium sulfate
mg: Milligram
STAT: *Statim*

## References

[1] Yıldırım E. Spontaneous triplet pregnancy and trap sequence, case report. BMC Pregnancy Childbirth. 2019;19(1). 10.1186/s12884-019-2484-3 PMC6728985 31488101

[2] Triplet pregnancy—UpToDate [Internet]. [cited 2019 Nov 2]. Available from: https://www.uptodate.com/contents/triplet-pregnancy?search=multiplegestations&source=search_result&selectedTitle=6~150&usage_type=default&display_rank=6.

[3] Smith LK, Manktelow BN, Draper ES, Boyle EM, Johnson SJ, Field DJ. Trends in the incidence and mortality of multiple births by socioeconomic deprivation and maternal age in England: population-based cohort study. BMJ Open. 2014;4(4). 10.1136/bmjopen-2013-004514 PMC3987713 24699461

[4] Ghorbani M, Moghadam S. A triplet pregnancy with spontaneous delivery of a fetus at gestational age of 20 weeks and pregnancy continuation of two other fetuses until week 33. Glob J Health Sci [Internet]. 2015 Jun 11 [cited 2019 Nov 3];8(2):88–92. http://www.ncbi.nlm.nih.gov/pubmed/26383220. 10.5539/gjhs.v8n2p88 PMC4803941 26383220

[5] Dudenhausen JW, Maier RF. Perinatale Probleme von Mehrlingen. Deutsches Arzteblatt. 2010;107:663–8. 10.3238/arztebl.2010.0663 PMC2954517 20953254

[6] Wei J, Wu Q-J, Zhang T-N, Shen Z-Q, Liu H, Zheng D-M, *et al.* Complications in multiple gestation pregnancy: a cross-sectional study of ten maternal-fetal medicine centers in China. Oncotarget [Internet]. 2016 May 24 [cited 2019 Nov 3];7(21):30797–803. http://www.ncbi.nlm.nih.gov/pubmed/27127170. 10.18632/oncotarget.9000 PMC5058718 27127170

[7] Goodnight W, Newman R. Optimal nutrition for improved twin pregnancy outcome. Obstetrics Gynecol. 2009;114:1121–34. 10.1097/AOG.0b013e3181bb14c8 20168116

[8] Bhandari S, Ganguly I, Agrawal P, Bhandari S, Singh A, Gupta N. Comparative analysis of perinatal outcome of spontaneous pregnancy reduction and multifetal pregnancy reduction in triplet pregnancies conceived after assisted reproductive technique. J Hum Reprod Sci [Internet]. [cited 2019 Nov 3];9(3):173–8. http://www.ncbi.nlm.nih.gov/pubmed/27803585. 10.4103/0974-1208.192058 PMC5070399 27803585

[9] James S, Gil KM, Myers NA, Stewart J. Effect of parity on gestational age at delivery in multiple gestation pregnancies. J Perinatol [Internet]. 2009 Jan [cited 2019 Nov 3];29(1):13–9. http://www.ncbi.nlm.nih.gov/pubmed/18716629. 10.1038/jp.2008.121 18716629

[10] Udealor PC, Ezeome IV, Emegoakor FC, Okeke DO, Okere PCN. Delayed interval delivery following early loss of the leading twin. Case Rep Obstet Gynecol. 2015;2015:1–3. 10.1155/2015/213852 PMC4320913 25688311

[11] Multifetal pregnancy reduction and selective termination—UpToDate [Internet]. [cited 2019 Nov 3]. Available from: https://www.uptodate.com/contents/multifetal-pregnancy-reduction-and-selective-termination?search=multiplegestations&source=search_result&selectedTitle=4~150&usage_type=default&display_rank=4.

[12] Delayed-interval delivery in multifetal pregnancy—UpToDate [Internet]. [cited 2019 Nov 3]. https://www.uptodate.com/contents/delayed-interval-delivery-in-multifetal-pregnancy?search=multiplegestations&source=search_result&selectedTitle=7~150&usage_type=default&display_rank=7.

[13] Reinhard J, Reichenbach L, Ernst T, Reitter A, Antwerpen I, Herrmann E, *et al.* Delayed interval delivery in twin and triplet pregnancies: 6 years of experience in one perinatal center. J Perinat Med [Internet]. 2012 Sep [cited 2021 Apr 19];40(5):551–5. https://pubmed.ncbi.nlm.nih.gov/23104798/. 10.1515/jpm-2011-0267 23104798

[14] Arabin B, van Eyck J. Delayed-interval delivery in twin and triplet pregnancies: 17 years of experience in 1 perinatal center. Am J Obstet Gynecol [Internet]. 2009 [cited 2021 Apr 19];200(2):154.e1-154.e8. https://pubmed.ncbi.nlm.nih.gov/19110229/. 10.1016/j.ajog.2008.08.046 19110229

[15] Adegbite AL, Ward SB, Bajoria R. Perinatal outcome of spontaneously conceived triplet pregnancies in relation to chorionicity. Am J Obstet Gynecol [Internet]. 2005 Oct [cited 2019 Nov 3];193(4):1463–71. http://www.ncbi.nlm.nih.gov/pubmed/16202741. 10.1016/j.ajog.2005.02.098 16202741

[16] Downing M, Sulo S, Parilla B. Perinatal and neonatal outcomes of triplet gestations based on chorionicity. Am J Perinatol Rep. 2017;07(01):e59-63. 10.1055/s-0037-1599124. PMC5373902 28367358

[17] Temur I. A twin pregnancy provided with ICSI, an abortion of the first fetus at the 18th week and live birth of the second fetus at the end of the 36th week: a case report and literature review. J Matern Fetal Neonatal Med [Internet]. 2013 Sep [cited 2019 Nov 3];26(13):1355–8. http://www.ncbi.nlm.nih.gov/pubmed/23488587. 10.3109/14767058.2013.784253 23488587

